# Pharmacological depletion of microglia and perivascular macrophages prevents Vascular Cognitive Impairment in Ang II-induced hypertension

**DOI:** 10.7150/thno.44394

**Published:** 2020-07-25

**Authors:** Danielle Kerkhofs, Britt T. van Hagen, Irina V. Milanova, Kimberly J. Schell, Helma van Essen, Erwin Wijnands, Pieter Goossens, W. Matthijs Blankesteijn, Thomas Unger, Jos Prickaerts, Erik A. Biessen, Robert J. van Oostenbrugge, Sébastien Foulquier

**Affiliations:** 1 Department of Neurology, Maastricht University Medical Center, 6202AZ, Maastricht, The Netherlands.; 2 Department of Pathology, Maastricht University, Maastricht, 6200MD, The Netherlands.; 3 Department of Psychiatry and Neuropsychology, Maastricht University, 6200MD, Maastricht, The Netherlands.; 4 Department of Pharmacology and Toxicology, Maastricht University, 6200MD, Maastricht, The Netherlands.; 5 CARIM - School for Cardiovascular Diseases, Maastricht University, 6200MD, The Netherlands.; 6 MH&Ns - School for Mental Health and Neuroscience, Maastricht University, 6200MD, The Netherlands.; 7 IMCAR - Institute for Molecular Cardiology Research, RWTH Aachen, 52074, Germany.

## Abstract

**Rationale:** Hypertension is a major risk factor for cerebral small vessel disease, the most prevalent cause of vascular cognitive impairment. As we have shown, hypertension induced by a prolonged Angiotensin II infusion is associated with increased permeability of the blood-brain barrier (BBB), chronic activation of microglia and myelin loss. In this study we therefore aim to determine the contribution of microglia to hypertension-induced cognitive impairment in an experimental hypertension model by a pharmacological depletion approach.

**Methods:** For this study, adult* Cx3Cr1*^gfp/wt^x*Thy1*^yfp/0^ reporter mice were infused for 12 weeks with Angiotensin II or saline and subgroups were treated with PLX5622, a highly selective CSF1R tyrosine kinase inhibitor. Systolic blood pressure (SBP) was measured via tail-cuff. Short- and long-term spatial memory was assessed during an Object Location task and a Morris Water Maze task (MWM). Microglia depletion efficacy was assessed by flow cytometry and immunohistochemistry. BBB leakages, microglia phenotype and myelin integrity were assessed by immunohistochemistry.

**Results:** SBP, heart weight and carotid pulsatility were increased by Ang II and were not affected by PLX5622. Short-term memory was significantly impaired in Ang II hypertensive mice, and partly prevented in Ang II mice treated with PLX5622. Histological and flow cytometry analysis revealed almost complete ablation of microglia and a 60% depletion of brain resident perivascular macrophages upon CSF1R inhibition. Number and size of BBB leakages were increased in Ang II hypertensive mice, but not altered by PLX5622 treatment. Microglia acquired a pro-inflammatory phenotype at the site of BBB leakages in both Saline and Ang II mice and were successfully depleted by PLX5622. There was however no significant change in myelin integrity at the site of leakages.

**Conclusion:** Our results show that depletion of microglia and PVMs, by CSF1R inhibition prevents short-term memory impairment in Ang II induced hypertensive mice. We suggest this beneficial effect is mediated by the major decrease of pro-inflammatory microglia within BBB leakages. This novel finding supports the critical role of brain immune cells in the pathogenesis of hypertension-related cognitive impairment. An adequate modulation of microglia /PVM density and phenotype may constitute a relevant approach to prevent and/or limit the progression of vascular cognitive impairment.

## Introduction

Cerebral small vessel disease (cSVD) is an age-related cerebral microangiopathy 1, 2. It is expected that the prevalence of cSVD will increase in our aging society 2, 3. cSVD is the leading cause of vascular cognitive impairment (VCI), an umbrella term that covers all cognitive disorders from mild cognitive impairment to vascular dementia 1, 4. Hypertension is the major risk factor for the development of cSVD^2^. cSVD is associated with structural abnormalities on brain magnetic resonance imaging (MRI) including lacunes, white matter hyperintensities (WMH), cerebral microbleeds and enlarged perivascular spaces 5. Despite a profound impact on human health, there is no specific treatment for cSVD 6, 7, mainly due to limited understanding of the disease's pathobiology.

There is increasing evidence that blood-brain barrier (BBB) dysfunction plays a pivotal role in the pathophysiology of cSVD 8-12. Under healthy conditions, the BBB functions in a well-regulated manner to ensure the provision of nutrients while protecting brain cells from blood constituents by forming a physical barrier 10, 13, 14. The BBB is composed of vascular endothelial cells interconnected through tight junctions, flanked by pericytes, astrocytes, microglia cells and perivascular extracellular matrix. Proper interaction between its cellular and noncellular components is required to maintain a selective barrier function of the BBB 13, 14. MRI studies have demonstrated higher BBB permeability in cSVD patients 8, 9 and this was associated with structural brain damage and cognitive impairment 8, 9, 15. Pathological investigations have revealed the association of BBB leakages with WMH lesions and dementia 16, 17. Leakage of plasma components into the parenchyma will elicit a local inflammatory response by Fc receptor-induced microglia activation, amongst others 18-20. Microglia are brain resident myeloid cells which for their maintenance throughout the entire lifespan rely on their self-renewal capacity 21, 22, orchestrated by growth factor colony stimulating factor 1 (CSF1) and its receptor (CSF1R) 23. In order to maintain physiological conditions, microglia are critical 24-27 by responding to conditions of tissue damage notably by clearing the accumulated debris 28, 29. However, increased permeability of the BBB may lead to persistent microglia activation 30, 31, and potentially contribute to the progression of the pathology and especially white matter injury. Gaining insights into the BBB-microglia interplay appears therefore instrumental to understand cSVD pathology.

The use of Angiotensin II (Ang II) infusion has been previously associated with hypertension-induced cerebrovascular dysfunction including increased BBB permeability and neuroinflammation 32-34. We have also shown earlier the presence of activated microglia in association with BBB leakages and short-term memory impairment in a prolonged Ang II infusion (12 weeks) model 35. We hypothesize that the depletion of microglia protects against hypertension-induced cognitive dysfunction due to the absence of microglial activation at the site of BBB leakages. In this present study we aim to decipher the contribution of microglia to hypertension-induced cognitive impairment via their depletion using a highly selective CSF1R tyrosine kinase inhibitor.

## Material and Methods

### Animals

**Figure 1:**
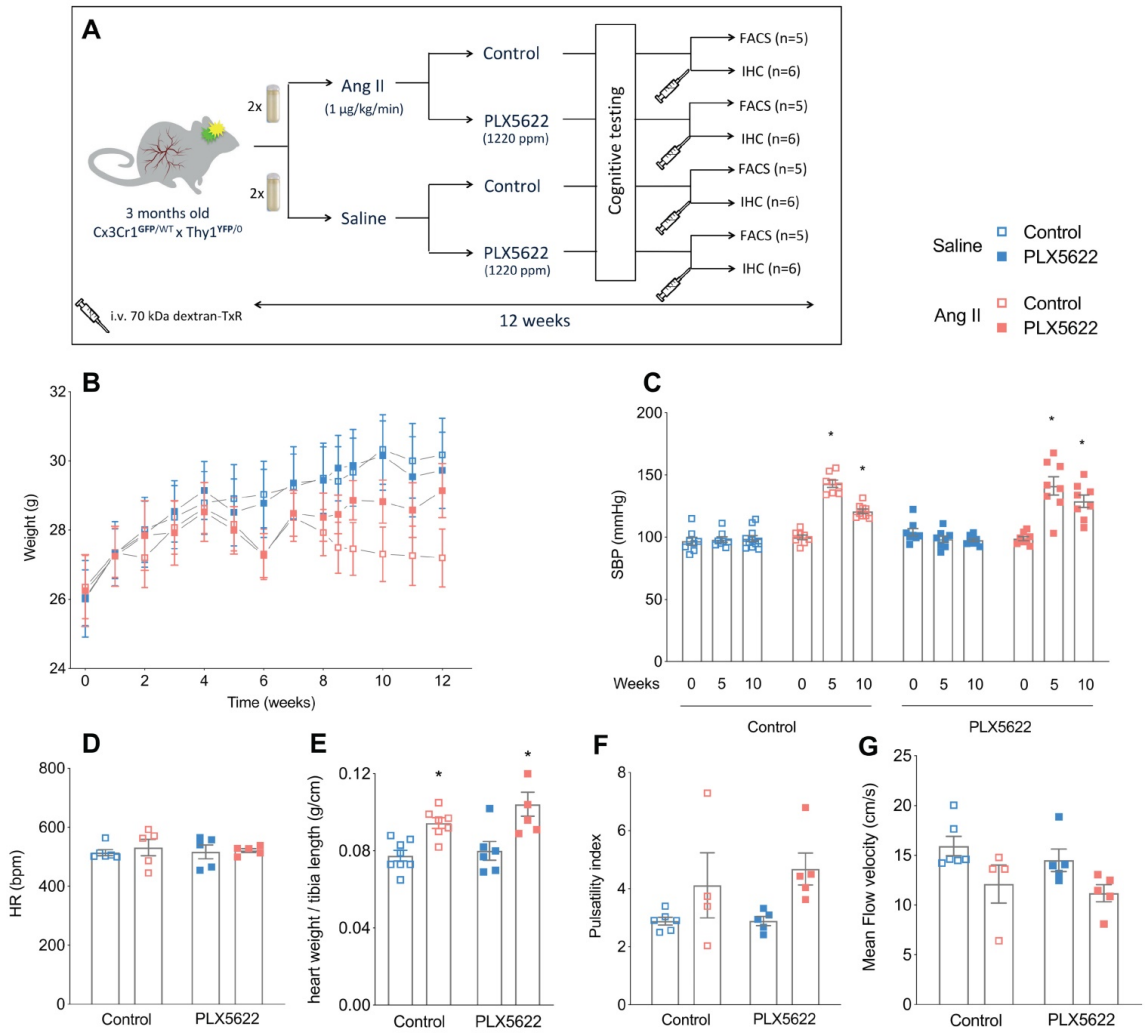
** Study design, body weight and cardiovascular parameters.** (**A**) Design of the study. (**B**) Body weight progression over the study period (2-W ANOVA p_int_ >0.05; p_time_< 0.001; p_groups_ < 0.001; n=9-11/group). (**C**) Systolic blood pressure values at baseline (week 0), mid-term period (week 5) and final-term period (week 10) (2-W ANOVA p_int_ < 0.001; p_time_< 0.001; p_AngII_ < 0.05; Tukey's multiple comparison test: *p < 0.05 vs. Saline; n=9-11 per group). (**D**) Heart rate at week 12 (2-W ANOVA p_int_ > 0.05; p_plx5622_ > 0.05; p_AngII_ >0.05; n=5-6 per group). (**E**) Cardiac hypertrophy (heart weight/tibia length) (2-W ANOVA p_int_ > 0.05; p_PLX5622_ > 0.05; p_AngII_ < 0.001; Tukey's multiple comparison test: *p < 0.05 vs. Saline; n=5-6 per group). (**F**) Carotid pulsatility index at week 12 (2-W ANOVA p_int_ > 0.05; p_plx5622_ > 0.05; p_AngII_ = 0.01; n=4-6 per group). (**G**) Mean Flow Velocity at week 12 (2-W ANOVA p_int_ > 0.05; p_plx5622_ > 0.05; p_AngII_ < 0.01; n=4-6 per group). (FACS: Fluorescence Assisted Cell Sorting; IHC: immunohistochemistry).

All animal experiments were approved by the regulatory authority of Maastricht University and were performed at Maastricht University in compliance with the national and European guidelines. *Cx3Cr1*^GFP/GFP^ mice (Jackson Lab 005582) were crossed with *Thy-1*^YFP/0^ mice (Jackson Lab 003782) to generate *Cx3Cr1*^GFP/WT^ x *Thy1*^YFP/0^ mice (abbreviated Tg mice) for microglial and neuronal visualization. Animals were kept on a normal 12h day-night cycle. All mice were allowed access *ad libitum* to water and mouse chow. The time frame of the experiment is depicted in **Figure 1A**. At the start of the experiment, 3-months old male Tg mice were fed with either PLX5622 laced chow (Plexxikon; CSF1R tyrosine kinase inhibitor, Ki =5.9 nM; 1200 ppm) or control chow for 12 weeks. The dose administered was chosen based on previous successful depletion studies 36, 37. At the same time, all mice were equipped with osmotic minipumps (Alzet model 2006, Direct Corp., Cupertino, CA, USA) for the delivery of Ang II (1 µg/kg/min subcutaneously) or saline. Osmotic minipumps were not primed prior to implantation and therefore microglia depletion by PLX5622 treatment was initiated 2-3 days before the onset of hypertension by Ang II infusion. Osmotic minipumps were implanted *s.c.* under isoflurane anesthesia and were replaced after 6 weeks with new minipumps for a total delivery time of 12 weeks. In total, 45 mice were included in the 4 groups (11-12 per group). Three animals died or were sacrificed prior to the end of the study due to hemorrhage in the thoracic cage (Ang II, vehicle), peritonitis due to a missed injection (Ang II, PLX5622) and severe weight loss after blood pressure measurement (Saline, PLX5622). Mice used for histology received an *i.v.* injection of 70kDa-dextran Texas Red (100 µL; 2.5 mg/mL in sterile NaCl 0.9%, D1864, Thermo Fisher), while under isoflurane anesthesia, to detect the presence of BBB leaks by histology. The injected dextran was allowed to circulate for 3 min before mice were euthanized by exsanguination. A second series of mice was euthanized after blood collection and intra-cardiac perfusion with PBS and heparin for FACS analysis of blood and brain samples.

### Cardiovascular phenotyping

Systolic BP was monitored before minipump implantation and after 5 and 10 weeks in awake mice using tail-cuff plethysmography (CODA, Kent Scientific) as previously described 35. Before sacrifice, ECG and carotid blood flow velocity signals were acquired non-invasively from mice anaesthetized by isoflurane using ultrasound flow velocity Doppler (20 MHz probe, DFVS, Indus Instruments, Webster, TX, USA). Mice were laid down in supine position on a temperature-controlled ECG board (Rodent surgical monitor; Indus Instruments, Webster, TX, USA). Body temperature was monitored with a rectal probe and maintained at 37°C. Three blood flow velocity records were saved per carotid. Heart rate (HR, bpm) and blood flow signals were analysed offline using the Indus Instruments Doppler Signal Processing Workstation and were averaged per mouse. Carotid pulsatility index (PI) was calculated as: PI = [Peak systolic velocity - End diastolic velocity]/Mean velocity.

### Cognitive tests

Long-term spatial memory was tested in the Morris Water Maze (MWM) 38. Shortly, the swim tank was divided into four quadrants and a platform was located in a fixed location. A video camera automatically recorded the mice movements (via a tracking system EthoVision, Noldus). Mice were subjected to the following testing schedule for MWM: spatial navigation with acquisition (days 1-4) and a probe trial (day 5). In all testing procedures the mice were given four trials per day with an inter-trial interval of 10 minutes and using four different starting positions. Each trial started with the mouse facing the wall of the pool and ended when the mouse reached the platform, or after 60 s. At day 5, a probe trial was conducted in which the platform was removed from the tank and the mouse was allowed to swim in the tank for 60 s. The swimming distance to reach the platform (cm) during test trials as well as the swimming speed and the time spent in the target quadrant during the probe trial (seconds), were obtained by Ethovision.

Short- and long-term memories were assessed in the two weeks preceding sacrifice. Short-term spatial memory was tested using an object location task (OLT) at 1 h intertrial intervals 35. Briefly, this 2-trial task consisted of a learning (T1) trial and a test trial (T2). In T1, a set of two identical objects were placed symmetrically in the middle of a circular arena, which the mouse was allowed to explore freely for 4min. After a 1 h interval spent in their home cage, the mice were placed in the arena for T2; in this 4 min-trial, one of the objects (right or left) was moved to a different location (front or back), while all other stimuli were kept the same. Mice will spend more time exploring the moved object than the stationary object if they remember the previous location. The time spent exploring each object was scored manually on a computer by an experimenter blind to the experimental groups. Trials were excluded from the analysis when the total exploration time was inferior to 6 seconds. The discrimination index d2 assesses whether the mouse spends more time at the novel location than at the familiar location (the difference between location exploration times divided by the total exploration time). Functional spatial short-term memory is reflected by a d2 index higher than zero (both objects equally explored) 39.

### Blood and brain FACS

Heparinized whole blood was used for flow cytometry using the following antibody cocktail: CD45-PerCP (Clone 30-F11; Biolegend 103130), CD3-eFLUO450 (Clone 17A2; eBioscience 48-0032), NK1.1-PE (Clone PK136; BD557391), Ly6G-APC-CY7 (Clone 1A8; BD560600), CD11b PE-CY7 (Clone M1/70; BD552850), Ly6C-APC (Clone 1G7.G10; Miltenyi 130-093-136), CD19-APC-H7 (Clone 1D3, eBioscience 47-0193) and Siglec-F-PE (Clone E50-2440; BD552126). Further, cells from the whole brain were isolated for FACS. Anesthetized mice were perfused with PBS to remove the peripheral blood. Subsequently, brains were dissected, mechanically dissociated and digested with a collagenase mix (including collagenase IX, collagenase I, DNAse and RPMI-HEPES). Immune cells were separated using a Percoll-gradient and stained with CD45 PerCP (Clone 30-F11; Biolegend 103130), CD11c PE-Cy7 (Clone N418, eBioscience 25-0114)), F4/80 (Clone BM8; Biolegend 123116), CD11b BV510 (Clone M1/70; Biolegend 101245), CD3 BV421 (Clone 17A2; eBioscience 48-0032-82), CD19 BV421 (Clone 6D5; Biolegend 115538), Ly6G BV421 (Clone eBio927; eBioscience 48-3172), MHC II-APC-eFluor780 (Clone M5/114.15.2; eBioscience 47-5321-82). All samples were measured with a FACS-Canto II (BD Biosciences). Results were analysed with FACSdiva version 8 (BD Biosciences).

### Immunohistochemistry

Brains were post-fixed with 4% paraformaldehyde overnight at 4ºC, washed with Tris Buffered Saline (TBS) and stored in TBS containing 0.1% sodium azide at 4ºC. Coronal sections (thickness 50 µm) were prepared using a vibratome (VT1200S, Leica). Free-floating sections were thoroughly washed with 0.3% Triton-TBS followed by antigen retrieval with target retrieval solution citrate pH6 (1:10, Dako S2031) for 20 minutes at 80ºC. After blocking with 1% of bovine serum albumin in 0.1% Triton-TBS, sections were incubated with primary antibodies anti-Iba1 (rabbit polyclonal; C-terminus Iba1; 1:1000; Wako 019-19741), anti-CD206 (MR5D3, rat polyclonal; CRD4-7-Fc protein; 1:200, Bio-Rad MCA2235), anti-CD68 (rat monoclonal, macrosialin protein; 1:500, bio-rad MCA1957), anti-Podocalyxin (rat monoclonal, Podocalyxin Ser21-Arg402; 1:100, R&D systemns MAB1556), anti-MBP (Myelin Basic Protein; a.a. 82-87; rat polyclonal, 1:1000 Millipore MAB 386) or anti-mouse IgG (Biotin-SP AffiniPure, donkey polyclonal, IgG (H+L); 1:100, Jackson ImmunoResearch N715-065-150) overnight at 4°C. After 3 washes, the sections were incubated with secondary antibodies including donkey-anti-rat biotin (Biotin-SP AffiniPure, donkey polyclonal, IgG (H+L); 1:400, Jackson ImmunoResearch 712-065-150) or donkey-anti rabbit biotin (Biotin-SP AffiniPure, donkey polyclonal, IgG (H+L); 1:400, Jackson ImmunoResearch 711-065-151), followed by conjugation with Streptavidin-AF647 (1:500, ThermoFischer S32357). Brain sections were mounted on gelatin-coated microscopic slides using fluorescence mounting medium (Dako S3023) and examined with a slide scanning microscope (Nikon Eclipse Ti-E) and a confocal microscope (Leica SPE).

### Image analysis

All analyses were performed by a blinded investigator using ImageJ (Fiji Distribution, NIH). Podocalyxin-positive brain capillaries were imaged within the cortex and corpus callosum (*x*=275; *y*=275; *z*=22 µm; n=5-6) The internal diameters of 10 capillaries within 2 randomly selected volumes per brain area were measured in Fiji and averaged per animal to assess the presence of a possible inward vascular remodelling. Microglia were counted as Cx3Cr1^+^/Iba1^+^ cells on 6 randomly selected volumes within the neocortex (*x*=366; *y*=366; *z*=20 µm; n=5-6). Microglia were considered pro-inflammatory based on their CD68 expression. The number of Cx3Cr1^+^ and Cx3Cr1+ CD68^+^ cells were counted on 4 randomly selected cortical volumes per animal (*x*=275; *y*=275; *z*=20 µm; n=5-6) and within BBB leakages (*x*= 175; *y*=175; *z*=10-20 µm; n=3-4). Soma size and ramifications length of cortical microglia were determined using WIS-NeuroMath software 40. Iba1 signal from 6 selected images was analysed using the following parameters: noise level, 5; measure level, cell morphology; segmentation type, threshold; minimal cell intensity, 90; minimal area, 20; maximal area, 1500; minimal diameter, 4; maximal axial ratio, 8; minimal neurite length, 5. Perivascular macrophages (PVMs) were counted as CD206^+^ cells^41^ on 8 volumes selected to include cortical penetrating vessels within the neocortex (*x*=275; *y*=275; *z*=22 µm; n=5-6). BBB permeability was assessed by determining the level of extravasation of the 70kDa-dextran probe and plasmatic IgG proteins into the brain parenchyma as shown previously 35. Identification of IgG leakages was performed morphometrically by one investigator, who was blinded to the experimental groups. BBB leakages were defined as a signal with an intense core and diffuse borders. A series of brain slices (6 slices per brain; n=6 mice; bregma +1,7 mm; +1,3 mm; +0,3 mm; -1,2 mm; -1,6 mm; -2,1 mm) were screened to identify and localize leakages of different sizes (>70 kDa using the injected dextran and > 150 kDa using the circulating IgG) (Nikon Ti-Eclipse slide scanner). Z-stack images of all identified leakages were then acquired by confocal microscopy (*x*=175; *y*=175; *z*=20 µm). Maximal intensity projection of the z stacks was performed per channel and the areas of the dextran and IgG signals were measured by ImageJ to quantify the leakage size in mm^2^. MBP signal was used to assess changes in myelin composition to study whether microglia depletion could reverse myelin loss within leakage areas. The intensity of the myelin signal was examined in 8 leakages versus contralateral brain regions without leakages. Image stacks (*x*=550; *y*=550; *z*=30 μm) of leakages were acquired by confocal microscopy. MBP signal was also used to assess the thickness of the corpus callosum, a myelin-rich area. In addition, MBP signal intensities were measured at 3 defined locations in the lateral and medial corpus callosum as well as within randomly selected volumes within the striatum (bregma level: +0.7 mm, 2 slices per brain, n=6).

### Chemicals

All chemicals were from Sigma-Aldrich (Zwijndrecht, The Netherlands) unless otherwise specified. PLX5622 was provided by Plexxikon Inc. and formulated in standard chow (AIN-76A).

### Statistical analysis

All statistical analyses were performed with GraphPad Prism 8 software. Data are expressed as mean ± standard deviation. The normality distribution was tested with the Shapiro-Wilk test. Two-way ANOVA tests were performed with Ang II and PLX5622 treatments as independent variables, followed by Tukey's or Sidak multiple comparison post-hoc tests between individual groups. A *p* value < 0.05 was considered as statistically significant.

## Results

### Ang II induces a hypertensive cardiovascular phenotype that is not altered by CSF1R inhibition

PLX5622 treatment had no impact on body weight progression. Ang II infusion decreased the body weight in both control and PLX5622 groups compared to Saline infused mice (27.2±0.8 g and 29.1±0.8 g in Ang II groups vs 30.2±1.1 g and 29.7±1.1 g in Saline groups respectively at week 12; **Figure 1B**, group effect *p*_Ang II_ < 0.05). Systolic blood pressure was increased by Ang II at 5 weeks in both PLX5622 and vehicle treated groups and remained elevated at 10 weeks in both groups (**Figure 1C**, *p*_Ang II_ <0.001), there was no influence of Ang II on the heart rate (**Figure 1D**). Heart weights, normalized to tibia lengths, as well as carotid pulsatility indices were significantly increased in the Ang II compared to Saline groups and not changed by PLX5622 treatment (**Figure 1E**, *p*_AngII_ <0.001 and **Figure 1F**, *p*_AngII_ <0.05, respectively). Similarly, the carotid mean flow velocity was reduced in Ang II compared to Saline groups and not altered by PLX5622 treatment (**Figure 1G**, *p*_AngII_ <0.05). The internal diameters of brain capillaries with the cortex and corpus callosum were unchanged by Ang II (**Figure S1A-C**).

### CSF1R inhibition depletes microglia and reduces the number of perivascular macrophages and circulating immune cells

**Figure 2:**
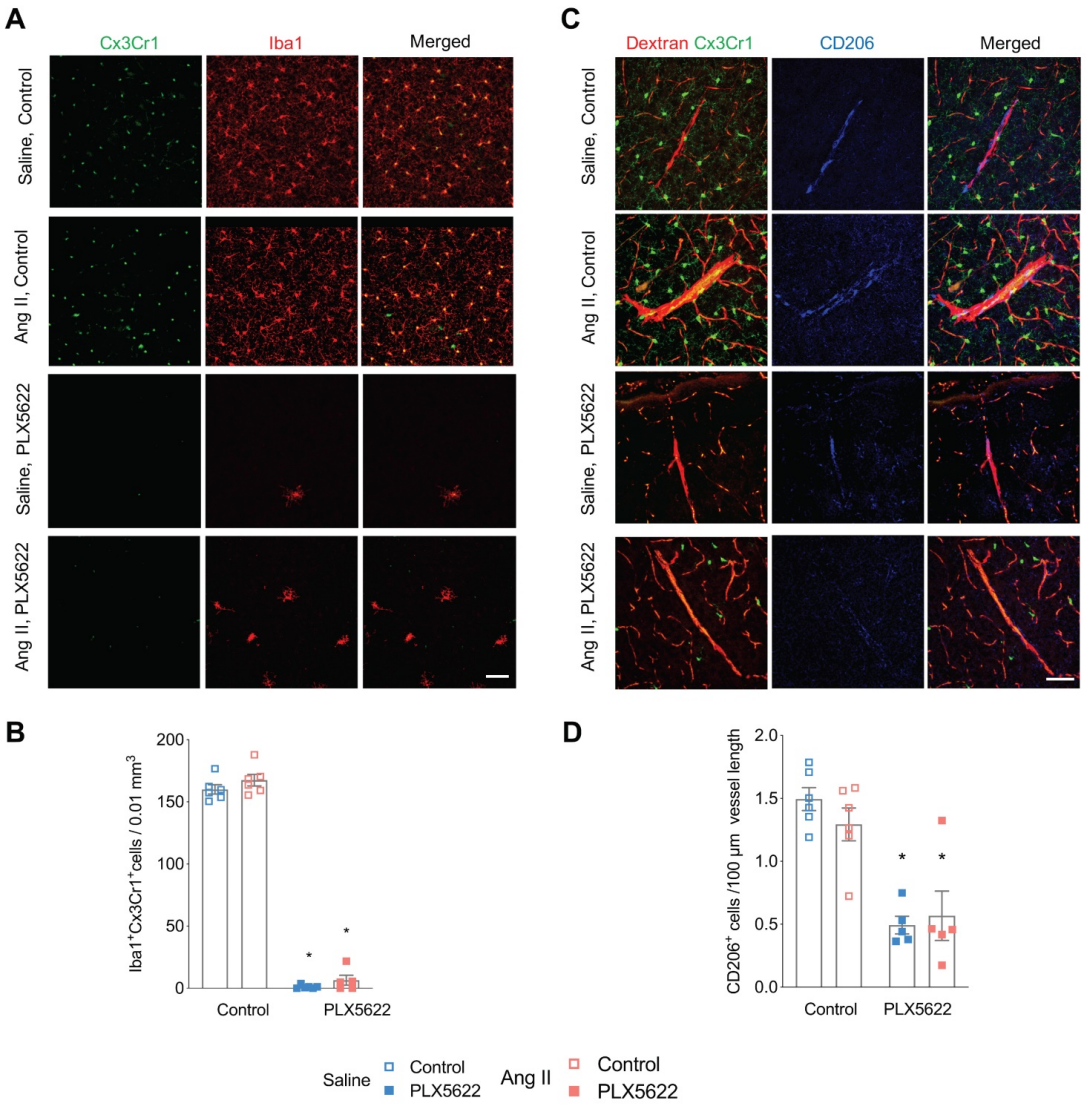
** Impact of CSF1R inhibition on microglia and perivascular macrophage densities.** (**A**) Representative pictures of Cx3Cr1-positve cells (green) and Iba-1 postive cells (red) in cortical areas (20x magnification, scale bar = 50 µm). (**B**) Microglia densities in cerebral cortex (2-W ANOVA p_int_ >0.05; p_plx5622_ < 0.001; p_AngII_ = 0.02; Tukey's multiple comparison test: *p < 0.05 vs. Control). (**C**) Representative picture of CD206^+^ staining. Left column: of Cx3CR1^+^ cells (green) and dextran- TxR (red). Middle column CD206^+^ elongated cells along the wall of penetrating neocortical vessels (blue). Right column: composite image. (40x magnification, scale bar = 50 µm). (**D**) Perivascular macrophage densities in cerebral cortex (2-W ANOVA p_int_ >0.05; p_plx5622_ < 0.001; p_AngII_ > 0.05; Tukey's multiple comparison test: *p < 0.05 vs. Control). n=5-6 per group.

Flow cytometry analysis of CD45^int^Cx3Cr1^hi^CD11b^hi^ cells revealed effective depletion of microglia in whole brains by the CSF1R inhibitor PLX5622 (**Figure S2A-C**, -94% in Saline groups; -83% in Ang II groups; *p*_PLX5622_<0.0001). This was confirmed by immunohistochemistry showing profoundly decreased density of Cx3Cr1^+^Iba-1^+^ cells in the cerebral cortex of PLX5622 treated mice (**Figure 2A,B**, -99% in Saline groups; -92% in Ang II groups; *p*_PLX5622_<0.001). Remaining microglia in PLX5622 treated groups exhibited a profound morphological change (**Figure S3A**). Microglia in the PLX5622 treated groups have an increased soma size (*p*_PLX5622_<0.001) and ramification length (*p*_PLX5622_<0.001) compared to vehicle treated groups (**Figure S3B-C**). Numbers of PVMs, CD206^+^ elongated cells surrounding cortical penetrating arterioles (**Figure 2C**), were also reduced in the PLX5622 treated groups (**Figure 2D**, -67% in Saline groups; -56% in Ang II groups; *p*_PLX5622_<0.001).

**Figure 3:**
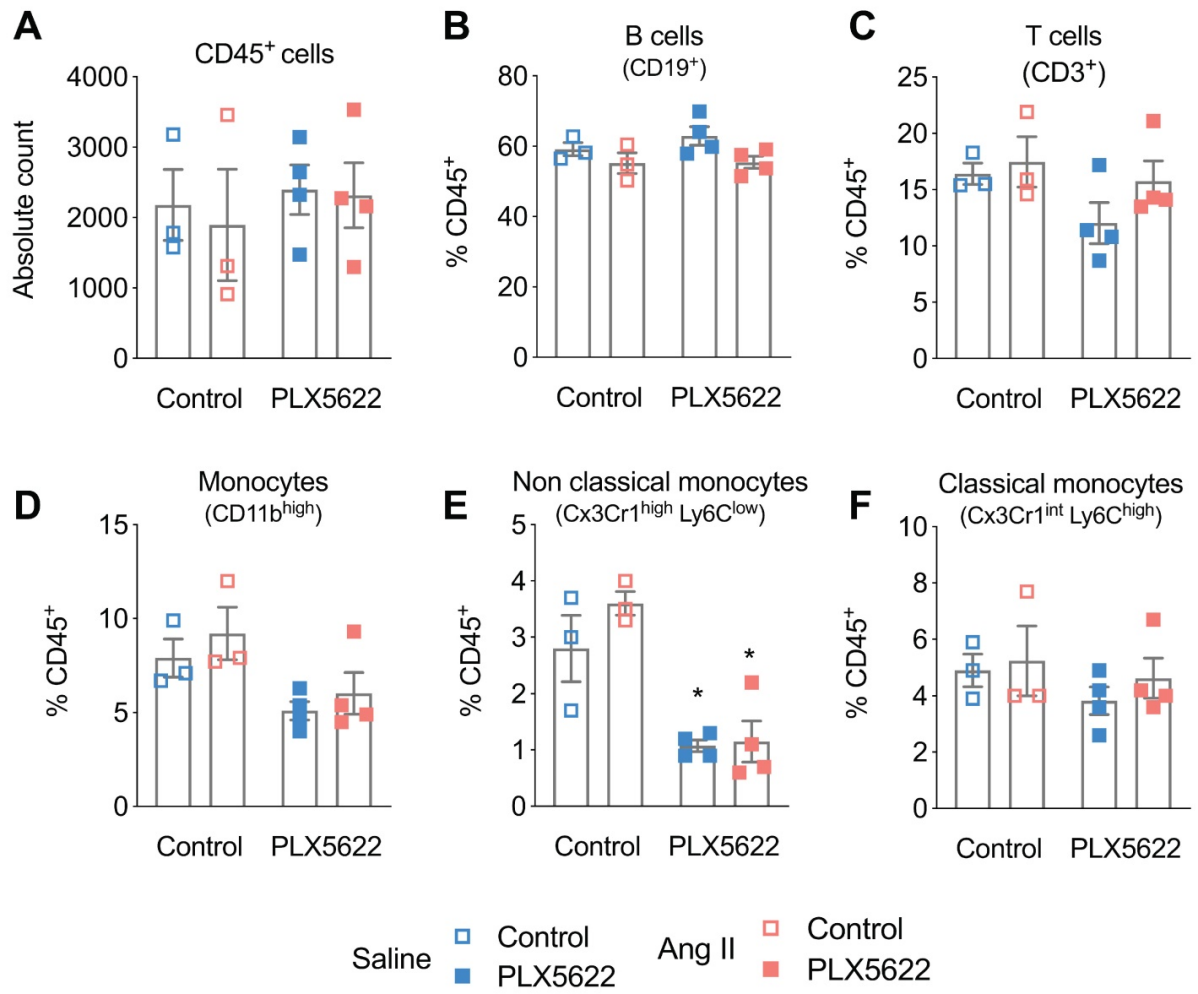
** Impact of CSF1R inhibition on circulating immune cells.** Cell counts of circulating (**A**) CD45^+^ cells, (**B**) CD19^+^ B-cells, (**C**) CD3^+^ T-cells, (**D**) monocytes, (**E**) of the Ly6C^low^ (**F**) and Ly6C^high^ subset, measured with flow cytometry. 2-way ANOVA; Tukey's multiple comparison post-test (*: p < 0.05 vs Vehicle). n=3-4 per group.

Treatment with PLX5622 did not influence CD45^+^ cells, CD19^+^ B-cells and CD3^+^ T-cells in the blood (**Figure 3A-C**). CD45^+^Cx3Cr1^+^Ly6C^low^ non-classical monocytes were reduced after 12 weeks of PLX5622 treatment (-64% in Saline group; -69% in Ang II group; p_PLX5622_<0.001), whereas the number of CD45^+^Cx3Cr1^+^Ly6C^high^ classical monocytes did not change (**Figure 3D-F**).

### CSF1R inhibition attenuates short-term memory impairment in hypertensive mice

**Figure 4:**
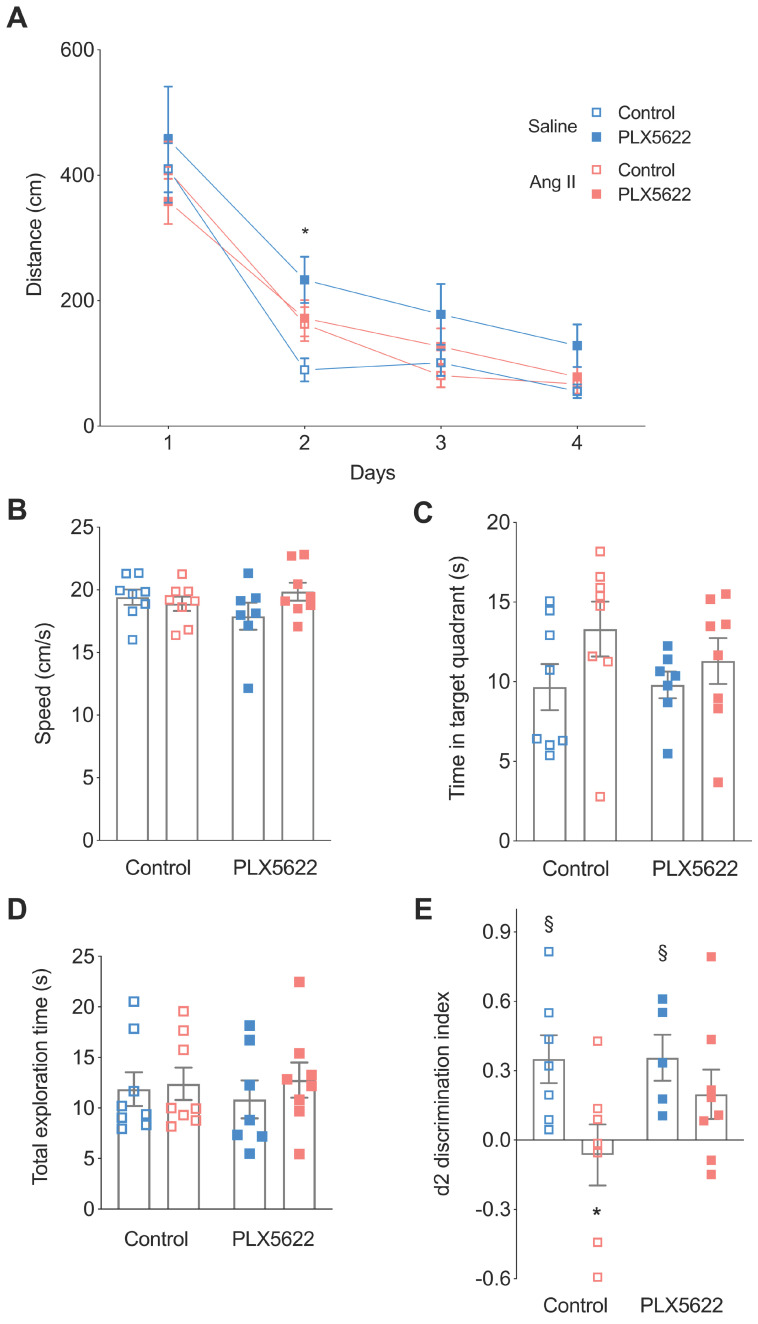
** Cognitive performance**. Long-term memory was assessed using the Morris Water Maze Task **(A,B,C)**. **(A)** Swimming distance to reach the platform (2-W ANOVA p_int_ >0.05; p_time_< 0.001; p_AngII_ < 0.05; Tukey's multiple comparison test: *p < 0.05 Saline, PLX5622 vs. Saline, Control). **(B)** Swimming speed (2-W ANOVA p_int_ > 0.05; p_plx5622_ > 0.05; p_AngII_ >0.05). **(C)** Time spent in target quadrant during the probe trial (2-W ANOVA p_int_ > 0.05; p_plx5622_ > 0.05; p_AngII_ >0.05). Short-term memory assessed using an Object Location Task at 12 weeks **(D,E)**. **(D)** Total exploration time (2-W ANOVA p_int_ > 0.05; p_plx5622_ > 0.05; p_AngII_ >0.05). **(E)** Discrimination index d2 (2-W ANOVA p_int_ > 0.05; p_PLX5622_ > 0.05; p_AngII_ < 0.05; Sidak's multiple comparison test: *p < 0.05 vs. Saline Control; Two tailed t-test: §: p <0.05 vs d2=0). n=7-9 per group.

Mice were first tested on the Morris water maze to evaluate learning and spatial memory 42. There was no overall effect of PLX5622 treatment and/or Ang II infusion on the escape distance during the training trials. The distance to reach the platform was however increased for the Saline PLX5622 group vs Saline controle group but only at day 2 (**Figure 4A**). For the probe trial, swimming speed was not different between the groups (**Figure 4B**), which is indicative of normal motor behaviour. Both Ang II and PLX5622 did not influence the time spent in the target quadrant (**Figure 4C**). Thus, long-term memory performance during the MWM task was not affected by Ang II and/or PLX5622 treatment.

In the object location task, the total exploration times did not differ between groups, indicating again that motor behaviour is normal in all animals (**Figure 4D**). Normotensive mice spent more time exploring the object at the novel location as indicated by the positive discrimination index d2 (Saline, Control: *p*=0.01; Saline, PLX5622: *p*=0.02). PLX5622 did not affect task performance in normotensive mice. Vehicle treated Ang II-infused mice were unable to discriminate between the two objects with d2 different from the Saline group and not different from zero, i.e. chance performance (*p*=0.69). However, Ang II-infused mice treated with PLX5622 performed significantly better compared to untreated Ang II-infused mice, as d2 did not differ from its respective Saline group. The discrimination index d2 was however not completely back to normal as it was still not statistically different from 0 (**Figure 4E**).

### Hypertension increased the number and size of blood-brain barrier leakages with no effect of the CSF1R inhibition

**Figure 5:**
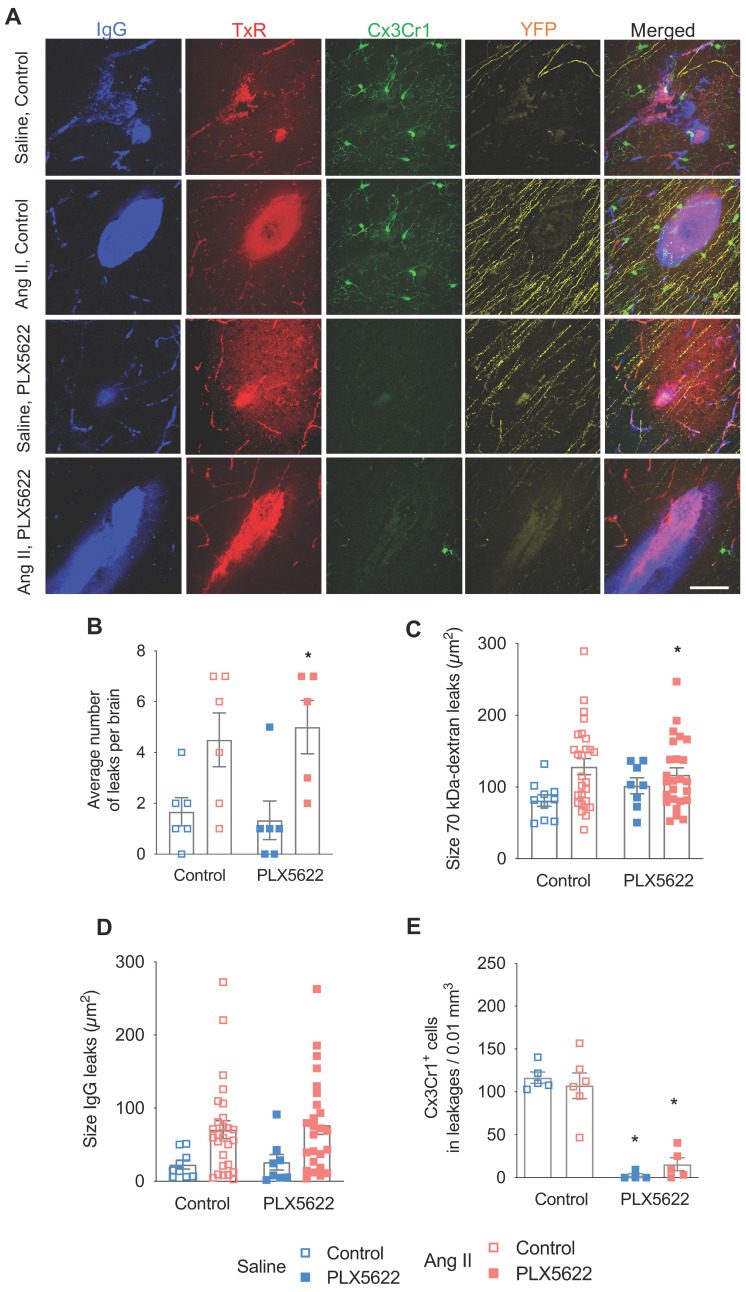
** Number and size of blood-brain barrier leakages. (A)** Representative pictures of leakages identified by IgG-positive (blue) and 70 kDa dextran-positive (red) signals, Thy-1^+^ axons (yellow) and Cx3Cr1^+^ cells (green) (scale bar = 50 µm). **(B)** Number of leakages per brain identiefied in 6 brain sections (2-W ANOVA p_int_ > 0.05; p_PLX5622_ > 0.05; p_AngII_ = 0.001; Sidak's multiple comparison test: *p < 0.05 vs. Saline). **(C)** Average size of the 70 kDa dextran leakages (2-W ANOVA p_int_ > 0.05; p_PLX5622_ > 0.05; p_AngII_ = 0.02). **(D)** Average size based of IgG^+^ leakages (2-W ANOVA p_int_ > 0.05; p_PLX5622_ > 0.05; p_AngII_ = 0.003). **(E)** Average density of Cx3Cr1^+^ cells in leakages per brain (2-W ANOVA p_int_ >0.05; p_plx5622_ < 0.001; p_AngII_ > 0.05; Sidak's multiple comparison test: *p < 0.001 vs. Control). n=5-6 per group.

BBB permeability was assessed by the extravasation into the brain parenchyma of the 70kDa-dextran probe and plasmatic IgG proteins (**Figure 5A**). BBB leakages were mainly distributed within the cerebral cortex (~40%), the hippocampus (~20%), the corpus callosum (~15%), the thalamus (~15%), and the striatum (~5%). Both the total number (**Figure 5B**, *p*_AngII_ = 0.001) and average size of BBB leaks, as judged from 70 kDa dextran (**Figure 5C**, *p*_AngII_ = 0.02) and IgG extravasation (**Figure 5D**, *p*_AngII_ = 0.003) were significantly increased in the Ang II groups. Treatment with PLX5622 did not influence number or size of BBB leaks. The local decrease in microglia density at the leakage site in the PLX5622 treated groups was equivalent to the observed global depletion (**Figure 5E**, *p*_plx5622_ < 0.001) and there was no effect of Ang II on the microglia density at the leakage sites.

### Microglia acquire a pro-inflammatory phenotype within blood-brain barrier leakages and are depleted by CSF1R inhibition with no improvement of myelin

**Figure 6:**
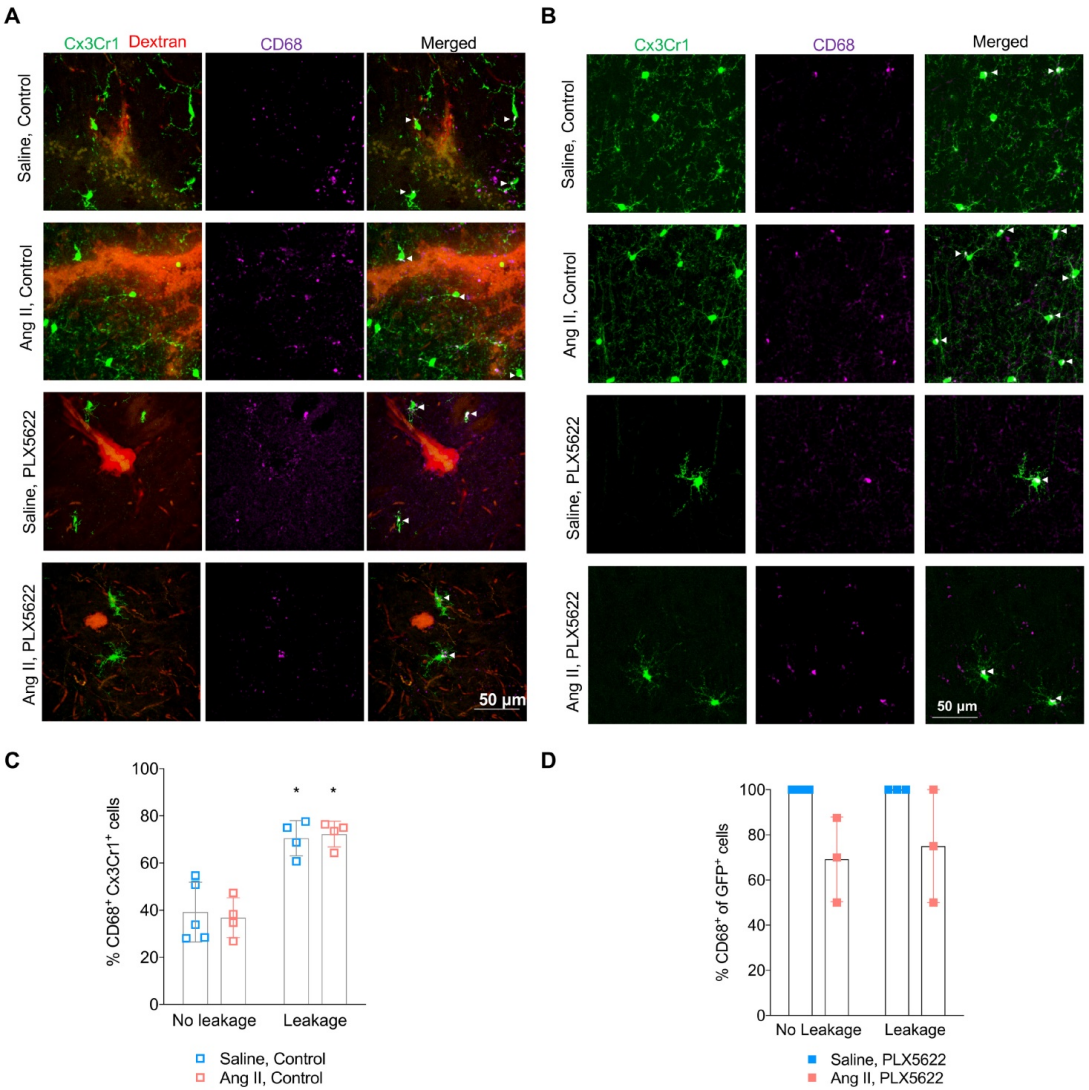
** Microglial activation in absence and presence of blood-brain barrier leakages. (A-B)** Representative pictures showing Cx3Cr1^+^ cells (green), CD68^+^ cells (purple) in presence **(A)** or absence **(B)** of a 70kDa dextran leakage (red) (scale bar = 50 µm). CD68^+^ Cx3Cr1^+^ activated microglia are indicated by white arrow heads. **(C)** Percentage of CD68^+^Cx3Cr1^+^ cells (leakage vs no leakage) for the vehicle treated mice (2-W ANOVA p_int_ > 0.05; p_leakage_ < 0.01; p_AngII_ > 0.05; Sidak's multiple comparison test: *p < 0.01 vs. No leakage). **(D)** Percentage of CD68^+^Cx3Cr1^+^ cells (leakage vs no leakage) for the PLX5622 treated mice (2-W ANOVA p_int_ > 0.05; p_leakage_ > 0.05; p_AngII_ < 0.05). n=3-5 per group.

**Figure 7:**
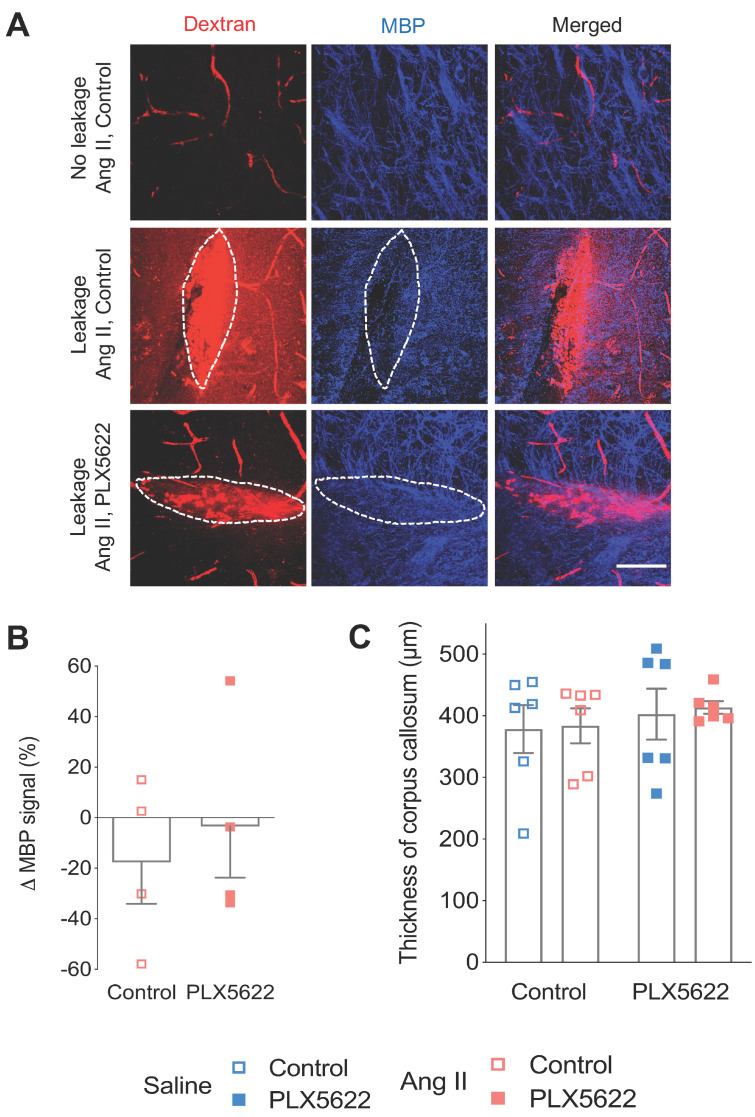
** Myelin loss at leakage sites. (A)** Myelin Basic Protein (MBP) signal (blue) in the absence (upper row) and presence (middle and botom row) of 70 kDa dextran leakages (red) in Ang II Control (middle row) and PLX5622 treated Ang II mice (bottom row) (63x magnification, scale bar = 50 µm). **(B)** Change of MBP signal in Dextran-positive vs Dextran-negative areas in Ang II mice treated with PLX5622 compared to Control; n=4 leakages per group.** (C)** Thickness of medial corpus callosum. n=6 per group.

Activated microglia expressing the CD68 marker were observed in presence and absence of BBB leakages (**Figure 6A,B**). The percentage of CD68^+^ Cx3Cr1^+^microglia was increased by the presence of BBB leakage for both the Saline and Ang II vehicle treated groups (**Figure 6C**, 71% vs 38% *p*_leakage_ < 0.01). While an effective reduction of Cx3CR1^+^CD68^+^ cells by PLX5622 was observed in the cortex (**Figure S4A-B**), the majority of the remaining microglia expressed CD68 (100% in Saline group and ~80% in Ang II group) independently of the presence/absence of a BBB leakage (**Figure 6D**). Myelin intensity in leakage sites in the PLX5622 treated animals was not significantly different between the saline and Ang II groups (**Figure 7A,B**, *p*=0.7). The size of the corpus callosum (**Figure 7C**) and the intensity (**Figure S5A-C**) of the myelin signal in corpus callosum and striatum were not different between the study groups.

## Discussion

An impaired BBB induced by chronic hypertension is associated with microglia activation and short-term memory impairment 32-35. In the present study we aimed to determine the contribution of microglia cells to hypertension-induced cognitive impairment using a pharmacological depletion approach. Our main finding is that short-term memory impairment caused by Ang II-induced hypertension was partly prevented after microglia / PVM depletion, in support of a critical role of brain resident immune cells in the pathogenesis of VCI. We used a chronic Ang II hypertension model in this present study which presents relevant features of cSVD as shown in our previous study 35 and in other studies 32, 33. We observed an impairment of the short term memory in the hypertensive non treated animal group in accordance with our previous study 35. The chronic Ang II hypertension was associated with a structural remodeling of the larger vessels (**Figure 1F-G**), a preserved lumen of the brain capillaries (**Figure S1**), and an increased number and size of BBB leakages (**Figure 5A-D**), an important feature of cSVD. Increase in systolic blood pressure has been shown to result in a reduction of cerebral blood flow and descreased cerebrovascular reactivity 43. To determine the effects of microglia/PVM depletion on cognitive function, short- and long-term spatial memory were assessed in an OLT and a MWM, respectively. In the OLT, we found that hypertensive mice had impaired short-term memory, as they could not discriminate between the new and the old object location, confirming earlier work by us 35, and others, based on similar tasks 33. Treatment with the CSF1R inhibitor PLX5622 partly obviated the hypertension associated impairment of short-term memory without altering the normal cognitive function of control mice. In the Morris Water Maze task however, both groups of Ang-II infused mice showed a normal spatial learning and memory as observed previously 44. The overall performance of the animals in the PLX5622 groups was also not altered, although post-hoc testing revealed an increased distance to reach the platform in the Saline PLX5622 group at day 2. It is debatable whether this statistical significant effect has any biological significance taking into account the overall lack of effect of PLX5622 on normal behaviour 36. One limitation inherent to the Morris Water Maze task is the use of water as an aversive stimulus since a differential anxiety can worsen a possible memory deficit. In our study, there was no effect on spatial learning and long-term memory in the MWM, suggesting no effect on anxiety although we did not test the anxiety behaviour of mice directly. In addition, we did not observe a change in the locomotor activity that could be induced by an increased/decreased anxiety (swimming speed, **Figure 4B**). In future studies, to minimize the possible impact of aversive stimuli on the animal's performance, we will favor the use of the Barnes Maze task.

The observed attenuated hypertension-induced short-term memory impairment mediated by CSF-1R inhibition is a novel finding. These results aligns with findings in other brain disease models as microglia depletion by CSF1R inhibition has proven to be effective to prevent radiation induced short-term memory impairment in mice 45 and to improve the cognitive function in a mouse model of Alzheimer's disease 36. In analogy to previous short- and long-term CSF1R inhibition studies, the overall behaviour and cognitive function of control mice were unaltered 23, 36, 45, 46. Furthermore, dampening microglial reactivity using minocycline, was beneficial for subcortical white matter functioning and cognitive performance in an animal model of chronic cerebral hypoperfusion 47, 48, strengthening the importance of targeting microglia to limit the impact of cerebrovascular diseases.

In accordance with our prior study 35, we found an increased BBB permeability in Ang II infused hypertensive mice, which was not altered by PLX5622 (**Figure 5B**). Increased BBB permeability causes leakage of plasma components which can induce microglia activation by binding the Fc receptors on these brain resident immune cells 18, 33, 49. We have observed an increased expression of the activation marker CD68 by microglia cells, at the site of BBB leakages compared to cortical areas without BBB leakage (**Figure 6C**). Quantification of the microglia density around the leakage sites revealed overt depletion of microglia cells surrounding the leakage in the PLX5622 treatment groups (**Figure 5E**), demonstrating that PLX5622 was also effective at the site of leakages, thereby dampening the neuroinflammatory response associated with leakages. It is of importance to note that most (80%-100%) of the very few microglia that escaped PLX5622 treatment, had a high CD68 expression and activated morphology (both in the cortex and within BBB leakages). This finding is in accordance with a previous study showing the expression of activation markers in proliferative (Ki67+) retinal microglia in the phase of repopulation 50.

Emerging evidence from animal models and clinical studies on cSVD points to a clear association between increased blood brain barrier permeability and cognitive impairment 11, 15, 30. Our results confirm the association between an increased BBB permeability and cognitive impairment in the untreated hypertensive group and moreover add new knowledge by demonstrating that CSF1R targeted depletion of microglia/PVM can prevent cognitive decline in hypertensive mice despite increased BBB permeability. While microglial reactivity following an acute BBB dysfunction is part of their physiological response as guardians of the CNS, we think however that a larger and recurrent BBB dysfunction due to the chronic exposure to risk factors (e.g. hypertension) can lead to local, exaggerated and sustained microglial reactivity with detrimental effects for cognitive functioning. Microglia depletion in an acute ischemic stroke model has shown to exacerbate stroke severity 51, while in our chronic VCI model, microglia/PVM depletion was able to prevent cognitive impairment. Although we could not associate the protective effect offered by the microglia depletion with an improvement of myelin intensity in sites of BBB leakages, we occasionally observed impaired neuronal tracts (Thy1^+^) at the site of leakages, which may account for the decline in short-term memory induced by Angiotensin II. The high variability of the locations and sizes of the BBB leakages is however not compatible with neuronal tracing of Thy1^+^ neurons. Furthermore, the observed beneficial effect of PLX5622 might also result from a preserved neuronal plasticity as a recent study has revealed that the downregulation of neuronal and synaptic genes in an Alzheimer's disease model was prevented in absence of microglia 52.

Microglia depletion can be achieved by genetic deletion (*Csf1r^-/-^ Pu.1^-/-^* mice), or by a pharmacologic depletion (CSF1R signaling inhibition) 29. In the present study, we opted for pharmacological depletion as it is time-controlled and applicable in any mouse strain, using the highly selective CSF1R inhibitor PLX5622 for the depletion of microglia cells. PLX5622 has proven to induce a rapid and effective microglia depletion of more than 90% after 3-7 days of treatment 36, 45, 53, 54. Even after 3 months of PLX5622 treatment cortical Iba-1^+^Cx3Cr1^+^ cell numbers were reduced by 90% (**Figure 2B**), a finding that was confirmed on whole brain by flow cytometry of CD45^int^Cx3Cr1^hi^CD11b^hi^ cells. These data are in line with previous findings using CSF1R inhibition in several mouse models 36, 55. While tissue macrophages and circulating monocytes express CSF1R as well, their survival is not only depending on CSF1/CSF1R signalling but relies also on CCL2/CCR2 signalling 56 which is not present in microglia. We expected therefore that the PLX5622 treatment would lead to a mild reduction of tissue macrophages and monocyte populations 57. Perivascular macrophages (PVMs) are brain macrophages that originate from hematopoietic precursors cells, express the mannose receptor (CD206^+^) and reside in the perivascular space of penetrating vessels 58, 59. Activation of PVMs subsequent to BBB leakage has been shown to induce the production of ROS 41, 60. Their depletion, using the injection of clodronate liposomes, was able to preserve both short-and long-term memory in a spontaneous hypertensive mouse model 41. Although there was no change in PVM numbers following Ang II infusion in our study, as observed in earlier work 41 we found however a 60% reduction of the CD206^+^ PVM numbers due to the PLX5622 treatment. We cannot exclude that the favorable cognitive outcome in the hypertensive group after PLX5622 treatment is partly due to a reduction in the number of PVMs, although the total number of PVMs, limited to the perivascular space of large vessels, is clearly inferior to the > 2 million microglia populating mouse brain 61. To further investigate the effect of long-term CSF1R inhibition on circulating immune cells, we performed flow cytometry of the peripheral blood. We found a reduction of more than 50% of the non-classical Ly6C^low^ monocytes in the PLX5622 treated groups while there was no effect of the treatment on the classical Ly6C^high^ monocytes. Comparable results were obtained in other studies 53, 62 and the selective partial depletion of non-classical monocytes is in line with their low/negative CCR2 expression in comparison to classical monocytes (CCR2^high^), making them vulnerable to CSF1R inhibition. There is in the literature no evidence that non-classical monocytes are directly involved in the pathogenesis of cerebrovascular disease or that depletion of the non-classical monocytes is protective in this disease.

Depletion of microglia/PVM as achieved in our study, did not lead to a blood pressure reduction. The contribution of both macrophage subsets in the modulation of the autonomic activity in cardiovascular regulatory centers is well known 35, 63-67. Previous studies reported that Ang II was able to increase blood pressure via the activation of microglia due to an increased BBB permeability in the paraventricular nucleus of the hypothalamus (PVN) in hypertensive animals 63, 68. We did not observe a decrease of BP after microglia /PVM depletion, possibly because cells were already depleted prior to, not after 68, Ang II-induced neurogenic hypertension. This is also largely due to the pleiotropic actions of Ang II on the cardiovascular system that suffice to induce the blood pressure elevation without its central contribution.

The use of the *Cx3Cr1*^GFP/WT^ x *Thy1*^YFP/0^ mouse model may have led to a reduction in Cx3Cr1 levels. As the insertion of GFP in the Cx3cr1^GFP^ mouse model was performed at the expense of 390 base pairs in the second exon of the Cx3Cr1 gene, its interaction with its ligand Cx3CL1 is altered as demonstrated when the mouse line was developed 69. As a result, only heterozygous mice - as in the present study - should be studied to understand the behaviour of microglia under healthy and pathological conditions 70.

In addition, although our study was designed to investigate the critical role played by microglia in the context of hypertension-induced cognitive dysfunction, we cannot exclude the possible contribution of other glial cells in the BBB dysfunction and cognitive impairment induced by Ang II. In particular, the activation of astrocytes has been evidenced in another study with Ang II hypertensive mice 71. Of note, the inhibition of CSF1R using PLX5622 has been shown to not alter the number of astrocytes nor oligodendrocytes 36. It would be of interest to investigate if the blockade of astrocyte reactivity alone or in combination with CSF1R inhibition could fully prevent the short-term memory impairment induced by Ang II.

In summary, we have shown that short-term memory impairment induced by prolonged Ang II infusion is partly prevented when microglia/PVM are depleted using a CSF1R inhibitor. Cognitive effects of PLX5622 treatment were independent of changes in cardiovascular function and blood brain barrier permeability. This novel finding supports the hypothesis that brain resident immune cells play a critical role in the pathogenesis of hypertension related cognitive impairment and further highlights the importance of the vascular-immune interplay for brain homeostasis. This is a major step towards the development of theranostics targeting the CSF1R (e.g. [^11^C]CPPC 72). It also provides pre-clinical evidence on the relevance and safety of CSF1R inhibition that may be advantageous compared to clodronate treatment. Further studies would be needed to develop a therapeutic scheme compatible with its administration in patients that would allow transient and safe microglia depletion in humans. An adequate modulation of microglia/PVM density and phenotype may constitute a relevant approach to prevent and/or limit the progression of vascular cognitive impairment. It remains however crucial to further decipher the molecular mechanisms by which microglia/PVMs can affect the metabolism and function of the neurovascular unit to improve our understanding of the pathogenesis of hypertension-induced VCI. This may include the imaging over time of inflammatory cytokines, reactive oxygen species and lipid peroxidation that may altogether provide a refined characterization of microglial/PVM function.

## Supplementary Material

Supplementary figures.

## Abbreviations

Ang II: Angiotensin II
BBB: blood-brain barrier
CSF1: colony stimulating factor 1
CSF1R: colony stimulating factor 1 receptor
cSVD: cerebral Small Vessel Disease
MBP: myelin basic protein
MRI: magnetic resonance imaging
MWM: Morris water maze
OLT: object location task
PVM: perivascular macrophages
SBP: systolic blood pressure
SEM: standard error of the mean
TBS: tris buffered saline
VCI: vascular cognitive impairment
WMH: white matter hyperintensities
