# Supplementary material for: Pharmacological depletion of microglia and perivascular macrophages prevents Vascular Cognitive Impairment in Ang II-induced hypertension
Source: Theranostics. 2020 Jul 25;10(21):9512–27. doi: 10.7150/thno.44394 (PMC7449902; doi:10.7150/thno.44394)
Supplement: Supplementary file 1 — Supplementary figures. [file thnov10p9512s1.pdf]

Pharmacological depletion of microglia and perivascular macrophages prevents vascular cognitive impairment in Ang II-induced hypertension

Danielle Kerkhofs[1,2,5], Britt T. van Hagen[3,6], Irina V. Milanova[4,5], Kimberly J. Schell[2,5], Helma van Essen[5], Erwin Wijnands[2,5], Pieter Goossens[2,5], W. Matthijs Blankesteijn[4,5], Thomas Unger[5], Jos Prickaerts[3,6], Erik A. Biessen[2,5,7], Robert J. van Oostenbrugge[1,5,6], Sébastien Foulquier[4,5,6]

Supplementary Figures

Figure S1: Internal diameters of podocalyxin-positive brain capillaries.

Figure S2: FACS analysis from whole brain homogenates.

Figure S3: Microglial morphological analysis.

Figure S4: Microglia activation in the cortex in absence of BBB leakage.

Figure S5: Myelin intensity.

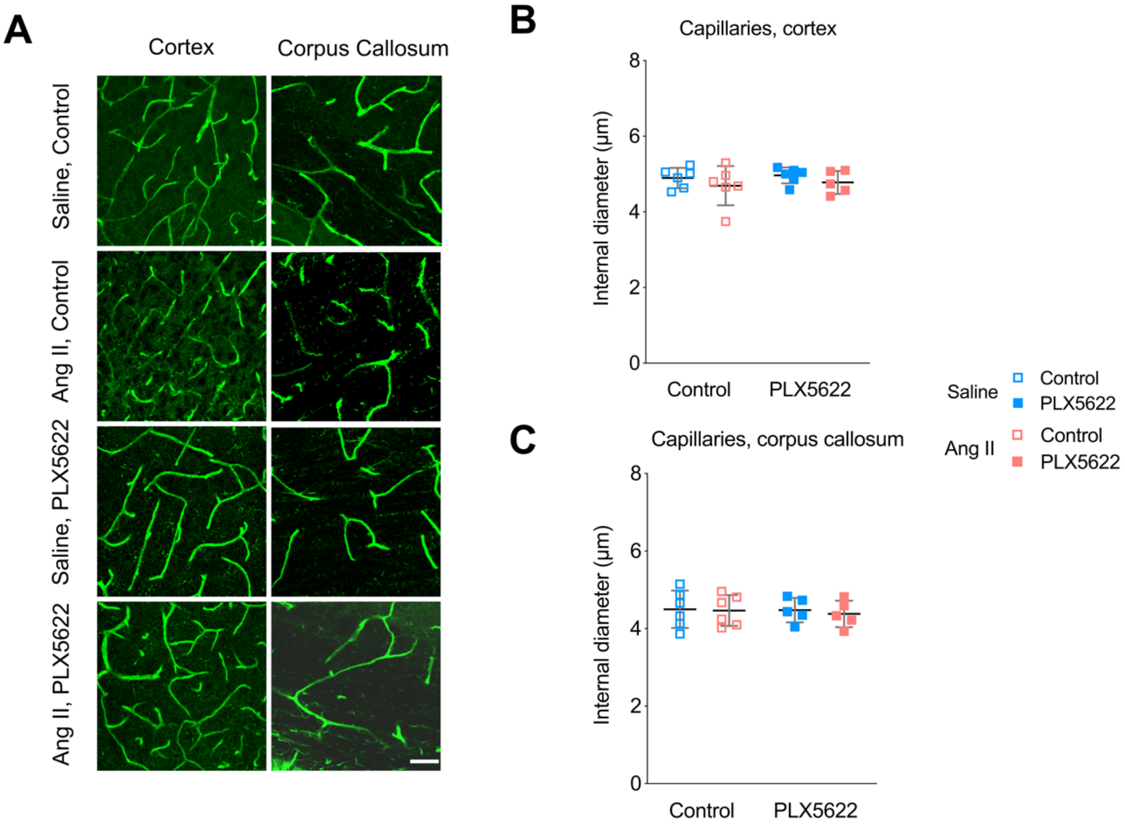

Supplementary Figure S1. Internal diameters of podocalyxin-positive brain capillaries. (A) Representative images of podocalyxin-positive capillaries (green) with the cortex and corpus callosum (scale bar = 50 µm). **(B)** Internal diameters of cortical capillaries (2-W ANOVA p_{int} >0.05; $p_{plx5622}$ >0.05; p_{AngII} > 0.05). **(C)** Internal diameters of capillaries in the corpus callosum (2-W ANOVA p_{int} >0.05; $p_{plx5622}$ >0.05; p_{AngII} > 0.05). n=5-6 mice per group.

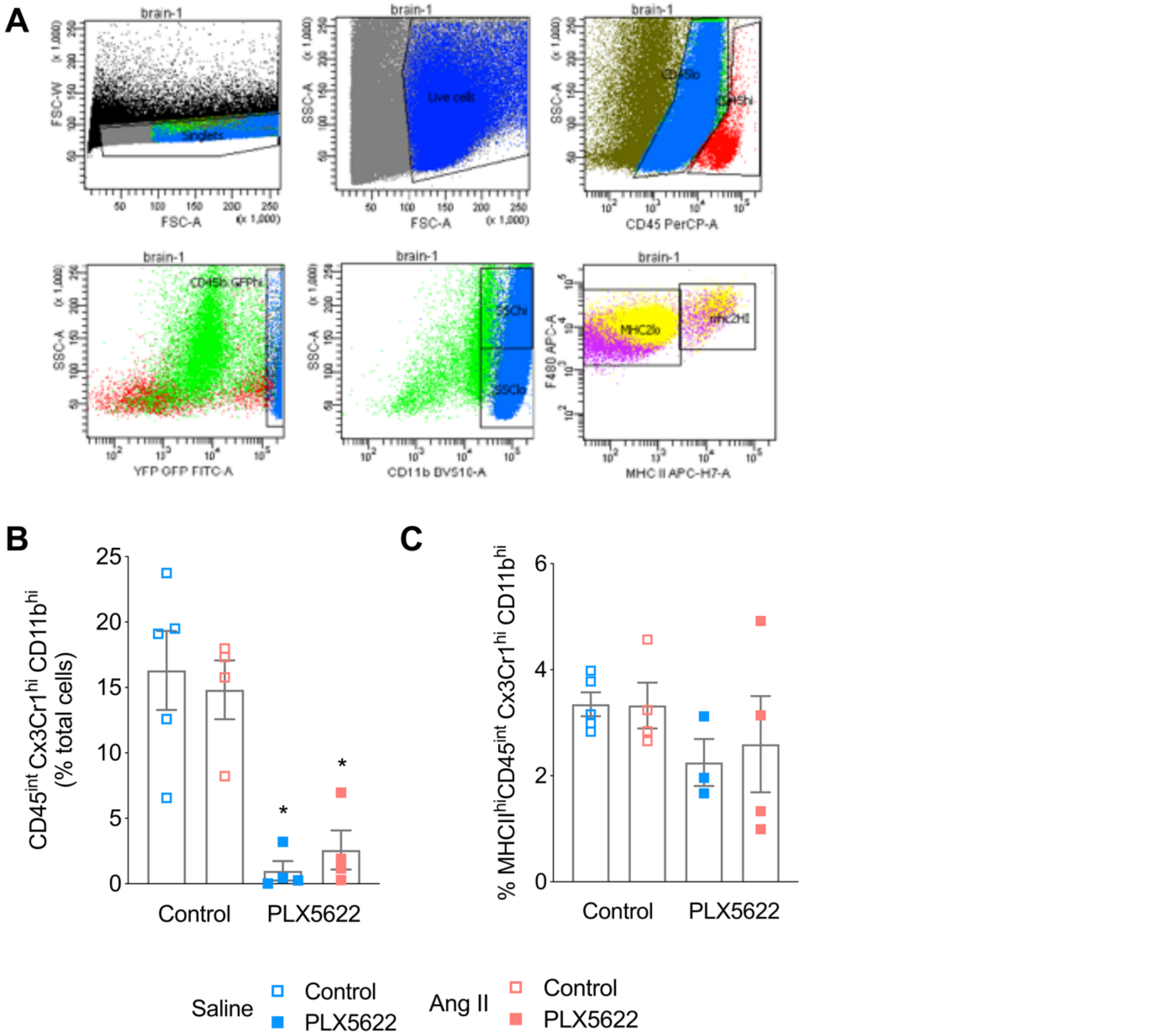

Supplementary Figure S2. FACS analysis from whole brain homogenates. **(A)** Representative flow cytometry gating of microglia population ($CD45^{int}$,$Cx3Cr1^{hi}$,$CD11b^{hi}$). **(B)** Proportion of microglia as percentage of total cells (2-W ANOVA $p_{int} > 0.05$; $p_{plx5622} < 0.001$; $p_{AngII} > 0.05$; Tukey's multiple comparison test: *p < 0.05 vs. control). **(C)** Proportion of $MHCII^{hi}$ microglia (2-W ANOVA $p_{int} > 0.05$; $p_{plx5622} > 0.05$; $p_{AngII} > 0.05$ (C). n=4-5 per group.

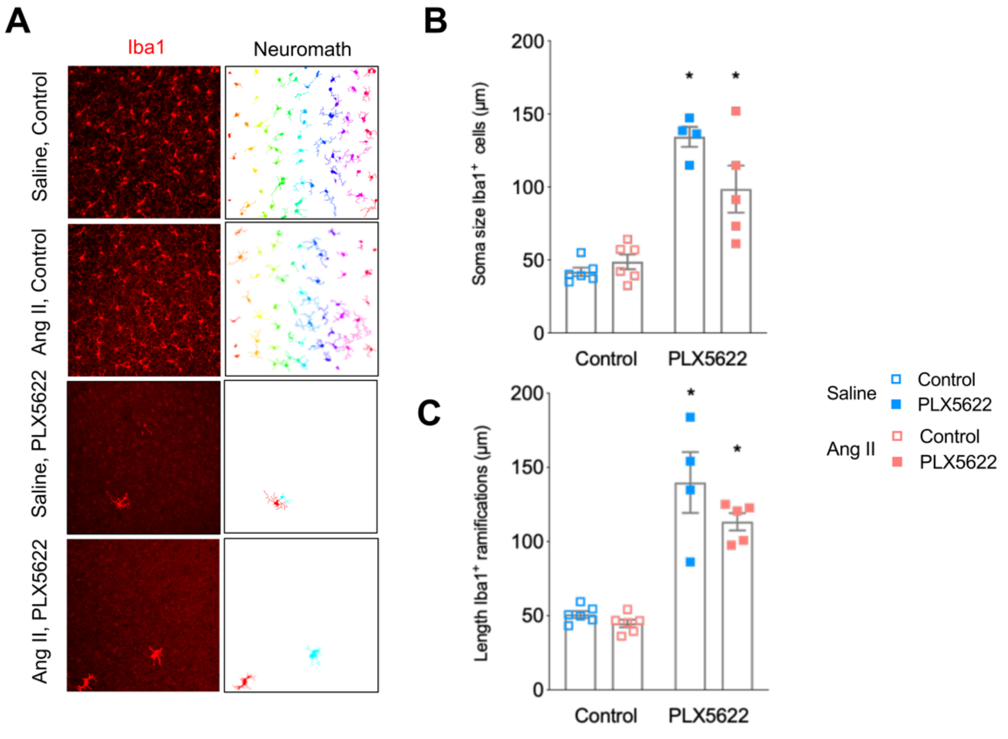

Supplementary Figure S3. Microglial morphological analysis. (A) Representative pictures of Iba1+ cells in cortical areas before (first column), and after automatic analyzes using WIS-NeuroMath software (second column) (scale bar = 50 µm). **(B)** Microglial cell soma size (2-W ANOVA $p_{int} = 0.027$; $p_{plx5622} < 0.001$; $p_{AngII} > 0.05$; Sidak's multiple comparison test: *p < 0.01 vs. control). **(C)** Microglial ramification lengths (2-W ANOVA $p_{int} > 0.05$; $p_{plx5622} < 0.001$; $p_{AngII} = 0.07$; Sidak's multiple comparison test: *p < 0.001 vs. control). n=5-6 per group.

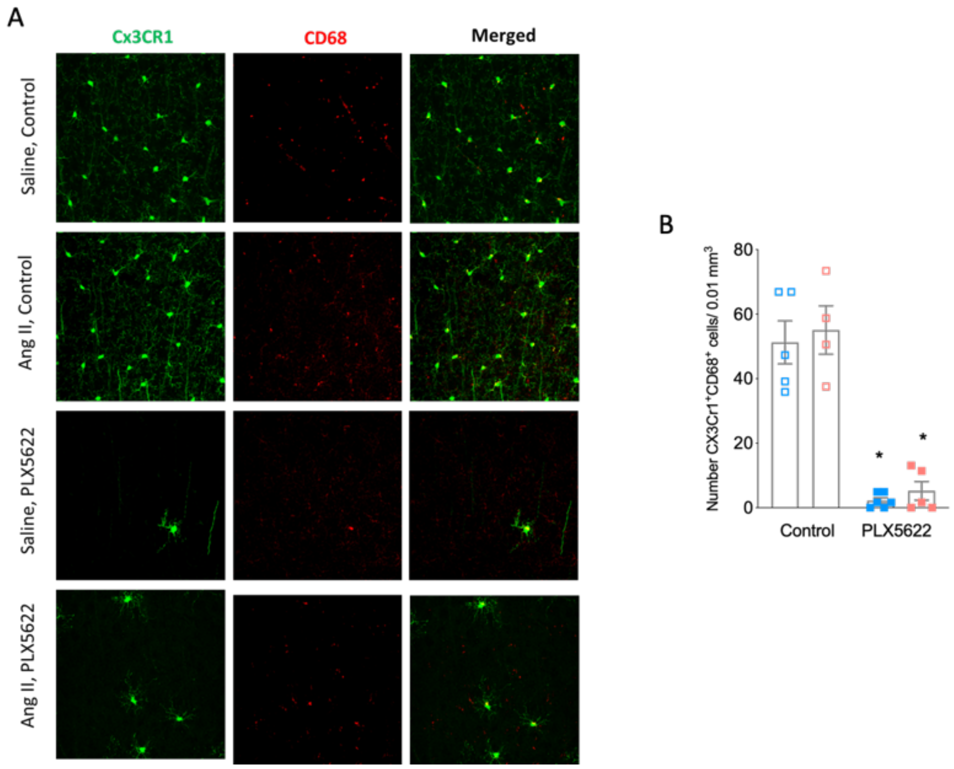

Supplementary Figure S4. Microglia activation in the cortex in absence of BBB leakage. (A) Representative pictures of Cx3Cr1$^+$ cells (green) and CD68$^+$ cells (red) (scale bar = 50 µm). **(B)** Cx3Cr1$^+$CD68$^+$ densities in cerebral cortex (2-W ANOVA $p_{int} > 0.05$; $p_{plx5622} < 0.001$; $p_{AngII} > 0.05$; Sidak's multiple comparison test: *p < 0.001 vs. Control). n=5-6 per group.

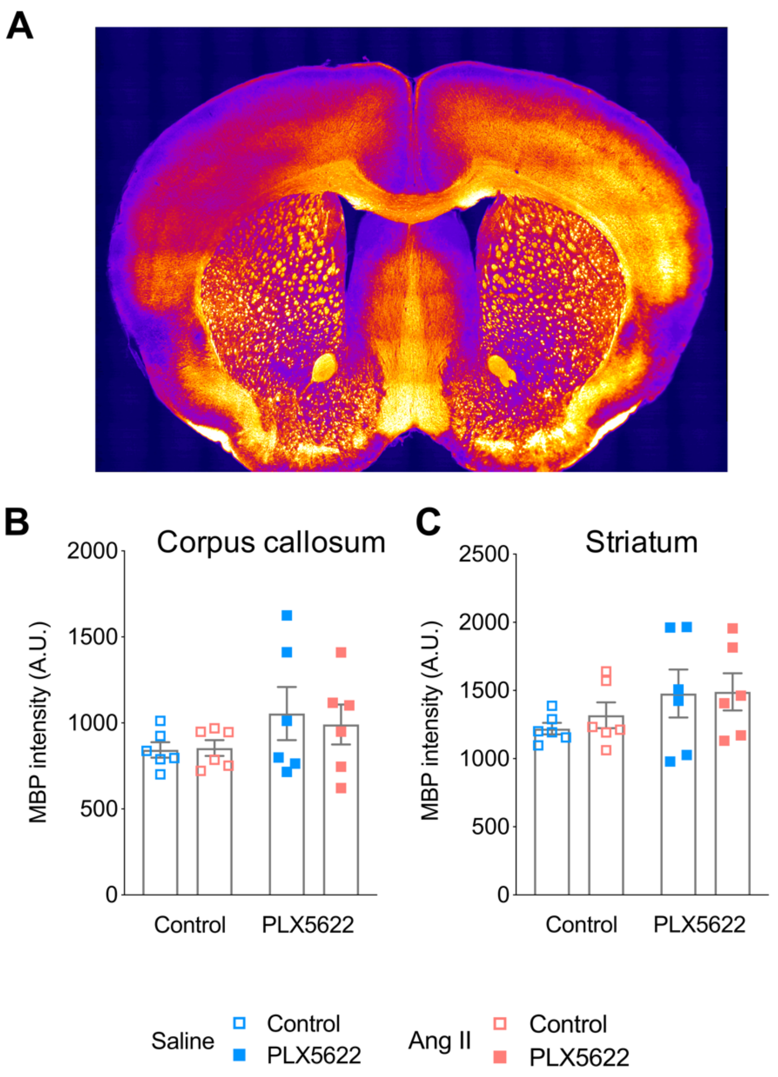

Supplementary Figure S5. Myelin intensity. (A) Representative Fire lookup table representation of a stitched coronal brain section stained for Myelin Basic Protein (MBP). **(B)** Average MBP intensity signal in corpus callosum (2-W ANOVA p_{int} >0.05; $p_{plx5622}$ > 0.05; p_{AngII} > 0.05). **(C)** Average MBP intensity signal in Striatum (2-W ANOVA p_{int} >0.05; $p_{plx5622}$ = 0.10; p_{AngII} > 0.05). n=6 per group.
